# Supplementary material for: Financial crisis early warning of Chinese listed companies based on MD&A text-linguistic feature indicators
Source: PLoS One. 2023 Sep 21;18(9):e0291818. doi: 10.1371/journal.pone.0291818 (PMC10513258; doi:10.1371/journal.pone.0291818)
Supplement: S1 Appendix — (DOCX) [file pone.0291818.s001.docx]

# Appendix

# Machine Learning Models

Machine learning methods can uncover the potential values and laws contained in the data to predict unknown data, so are widely used to solve complex problems in the field of engineering applications and science. This chapter will briefly introduce thirteen machine learning models.

# Logistic Regression, Ridge Regression, Lasso Regression

With linear regression as the theoretical support, logistic regression uses a Sigmoid function to map the results of the regression between 0 and 1 (without 0 and 1). The logistic regression model can be represented as the linear form in Equation (9).

| $\ln\frac{p_{xi}}{1-p_{xi}}=\beta_{0}+\beta_{1}X_{1}+\cdots+\beta_{n}X_{n}$ | (9) |
| --- | --- |

Where $p_{xi}$ represents the probability of the event occurring, $X_{i}$ denotes the explanatory variable, $\beta_{0}$ is a constant term, and $\beta_{1},\beta_{2},\cdots,\beta_{n}$ is the partial regression coefficient.

The overfitting problem of linear regression models can be better solved by adding a penalty term after the loss function of linear regression. There are two general types of penalty terms commonly used, which are L1 regularization and L2 regularization. The L2 regularization is used for ridge regression, and the L1 regularization is used for the least absolute shrinkage and selection operator (Lasso) regression.

# Support Vector Machine

Support vector machine (SVM) was first proposed by Vapnik [1]. Its learning strategy is to construct a segmentation hyperplane with maximum edge spacing between two classes of samples. The optimal classification hyperplane could be constructed by introducing a suitable kernel function, to achieve fast processing of high-dimensional inputs. In this paper, we use the mainstream radial basis kernel function, as shown in Equation (10).

| $k\left(x_{i},x_{j}\right)=\exp\left(-\frac{\left\Vert x_{i}-x_{j}\right\Vert^{2}}{2\sigma^{2}}\right)$ | (10) |
| --- | --- |

where $x_{i}$ and $x_{j}$ represent the feature vectors of the *i*-th sample and *j*-th sample, respectively. $\sigma$ represents the parameters of the radial basis kernel function.

# Back Propagation Neural Network

Back propagation neural network (BPNN) was proposed by Rumelhart et al. [2]. Its output results are propagated forward and the errors are propagated backward. This paper will use the ReLU function, which has sparsity so that it can make the sparse model better able to explore the relevant features and fit the training data. The specific function formula is shown in equation (11).

| $f(x)=\max(0,x)$ | (11) |
| --- | --- |

# Decision Tree

Decision tree (DT) is a tree-like structure that classifies samples based on features. The common DT generation algorithms are ID3, C4.5 and CART (Classification and Regression Tree) [3-5]. The ID3 and C4.5 algorithms are based on the information entropy model, which is computationally intensive; CART uses the Gini index as the index for classifying attributes in DT, which is conducive to simplifying the calculation. Therefore, the CART is chosen in this paper. $Gini\left(D\right)$ reflects the probability that two randomly selected samples from the dataset $D$ have inconsistent category labels. The smaller $Gini\left(D\right)$ is, the higher the purity of the dataset. The higher the "purity" of the DT nodes means that the samples contained in the branch nodes of DT are most likely to belong to the same category. The Gini index of the attribute $\alpha$ is expressed as equation (12), where V is the number of possible values of the attribute $\alpha$.

| $Gini(D,\alpha)=\sum_{v=1}^{V} \frac{\left\vert D^{v}\right\vert}{\vert D\vert}Gini\left(D^{v}\right)$ | (12) |
| --- | --- |

# Random Forest

Random forest (RF) consists of several DTs, and each DT is unrelated to the other. The majority of the classification results are chosen as the final result. Its final classification result can be shown as equation (13).

| $H\left(X^{B}\right)=\mathrm{argmax}\sum_{s=1}^{n} I\left(h_{s}\left(X^{B(s)}\right)=Y\right)$ | (13) |
| --- | --- |

where $h_{s}\left(X^{B(s)}\right)$ denotes the *s*-th DT model, $H\left(X^{B}\right)$ denotes the combined RF model, and *I*(·) is the schematic function.

# Gradient Boosted Decision Tree

Gradient boosted decision tree (GBDT) is proposed by Friedman [6]. This method firstly initializes the first base learner, then M base learners are established, and the negative gradient of the loss function is used as the residual approximation of the current DT to fit the new DT, and the formula for calculating the negative gradient is equation (14); after that, a value that reduces the loss as much as possible is found at the leaf nodes of the fitted tree; finally, the learners are updated.

| $r_{ti}=-\left[\frac{\partial L\left(y,f\left(x_{i}\right)\right)}{\partial f\left(x_{i}\right)}\right]_{f(x)=f_{t-1}(x)}$ | (14) |
| --- | --- |

where $r_{ti}$ denotes the negative gradient of sample i at the t-th iteration and $L\left(y,f\left(x_{i}\right)\right)$ represents the loss function, which can be expressed as Equation (15)

| $L(y,f(x))=\log(1+\exp(-yf(x))$ | (15) |
| --- | --- |

# CatBoost

CatBoost uses symmetric DT as the base model, and the processing process is as follows: firstly, the input sample set is randomly sorted and multiple sets of randomly arranged cases are generated; then the floating point type or attribute value tokens are transformed into integers; finally, all the results of category-type feature values are transformed into numerical results according to Equation (16).

|  | (16) |
| --- | --- |

where represents the indicator function, if the condition $\left(x_{j}^{i}=x_{k}^{i}\right)$ is satisfied, the function value takes 1, otherwise takes 0. $p$ is the prior value, and $\alpha$ is the weight of the prior value.

# XGBoost

XGBoost is an integrated algorithm based on CatBoost with a regularization term in the loss function, which integrates weak classifiers with low accuracy into a strong classifier with high accuracy. Its objective function is shown in equation (17).

| $obj(\theta)=\sum_{i}^{n} L\left(y_{i},\hat{y}_{i}\right)+\sum_{k=1}^{K} \Omega\left(f_{k}\right)$ | (17) |
| --- | --- |

Where $L\left(y_{i},\hat{y}_{i}\right)$ represents the loss function, $y_{i}$ represents the true value of the *i*-th sample, $\hat{y}_{i}$ represents the predicted value of the *i*-th sample, and $f_{k}$ represents the prediction function of the *k*-th tree.

# LightGBM

LightGBM has GBDT at its core and uses a one-sided gradient sampling algorithm, which maintains accurate information gain estimation, because the information gain is measured using the variance gain after splitting, retaining only those samples with larger contributions. The variance gain formula is given in equation (18).

| $\tilde{V}_{j}(d)=\left.\frac{1}{n}\left(\frac{\left(\sum_{x_{i}\in A_{l}} g_{i}+\frac{1-a}{b}\sum_{x_{i}\in B_{l}} g_{i}\right)^{2}}{n_{l}^{j}(d)}\right.+\frac{\left(\sum_{x_{i}\in A_{r}} g_{i}+\frac{1-a}{b}\sum_{x_{i}\in B_{r}} g_{i}\right)^{2}}{n_{r}^{j}(d)}\right)$ | (18) |
| --- | --- |

where *j* is the split feature used, *d* is the split point of the sample feature, *n* is the number of samples, *A* is the split large-gradient sample, *B* is the split small-gradient sample, *l* is the left subtrees, *r* is the right subtrees, and *g* is the sample gradient.

# AdaBoost

AdaBoost algorithm was first proposed by Freund & Schapire [7]. The training method focuses on updating the sample set weight values by each iteration. Finally, M weak classifiers are combined into a strong classifier according to their respective weights, as detailed in Equation (19).

| $G(x)=sign(f(x))=sign\left(\sum_{i=1}^{M} \alpha_{m}G_{m}(x)\right)$ | (19) |
| --- | --- |

where $G_{m}(x)$ is the *m*-th base classifier, the weight of which in the strong classifier is $\alpha_{m}$.

# Bagging

Bagging algorithm is a type of integrated learning method. First, the training set samples are drawn from the original dataset, which is with put-back sampling. One classifier is obtained using one training set at a time, and then all the classifiers obtained are voted on. The winner of its vote is the final classification result.

**References**

1. Vapnik VN. The Nature of Statistical Learning Theory. Springer Verlag, New York, USA; 1995.
2. Rumelhart DE, Hinton GE, Williams RJ. Learning representations by back-propagating errors. Nature. 1986;323:533–536. doi: 10.1038/323533a0.
3. Quinlan JR. Introduction of decision trees. Mach. Learn. 1986;1:84–100.
4. Quinlan JR. C4.5: Programs for Machine Learning. Morgan Kaufmann Publishers Inc., San Francisco, CA, USA; 1993.
5. Breiman LI, Friedman JH, Olshen RA, Stone C. Classification and regression trees (CART). Biometrics. 1984;40:358–361.
6. Friedman JH. Greedy function approximation: a gradient boosting machine. Ann. Stat. 2001;19:1189–1232.
7. Freund Y, Schapire RE. A decision-theoretic generalization of on-line learning and an application to boosting. J. Comput. Syst. Sci. 1997;55:119–139. doi: 10.1006/jcss.1997.1504.
