# Supplementary material for: Financial crisis early warning of Chinese listed companies based on MD&A text-linguistic feature indicators
Source: PLoS One. 2023 Sep 21;18(9):e0291818. doi: 10.1371/journal.pone.0291818 (PMC10513258; doi:10.1371/journal.pone.0291818)
Supplement: Attachment — Submitted filename: Response to Reviewer 2.doc [file pone.0291818.s003.doc]

Response to Reviewer 2

**Point 1:** Is this manuscript a review paper? There are no Innovation in methods.

**Response 1:** First, this manuscript is a research article, not a review paper. And then, Please allow me to make the following explanation:

Financial crisis early warning of listed companies usually starts with selecting some predictive indicators that are indicative of whether a financial crisis will occur in the future, and then based on these predictive indicators, applying statistical analysis or machine learning methods to predict the financial crisis. According to this process, the crisis early warning effect depends on two aspects: on the one hand, whether the predictive indicators can scientifically and reasonably provide a comprehensive evaluation of the overall situation of listed companies, and on the other hand, on the selection of forecasting method technology. In other words, the predictive indicators determine the upper limit of prediction accuracy, and the prediction method is to keep approaching this upper limit. Therefore, in terms of predictive indicators, the focus of this paper is to explore the incremental information of MD&A text-linguistic feature indicators for the financial crisis early warning of listed companies in order to further improve the upper limit of prediction accuracy; while in terms of methodology, although we did not innovate existing modeling methods, we explored the warning effects of 13 mainstream machine learning models, bridging the gap of multi-model comparative studies in this area.

**Point 2:** Although the manuscript is lengthy, there is relatively little original content created by the authors.

**Response 2:**

It is possible that our presentation in the manuscript was not clear enough to highlight our innovations. Therefore, we have added the shortcomings of existing studies and refined the contributions of this research in section "Introduction" as follows:

The main shortcomings of the existing studies are: (1) MD&A text tone and forward-looking information are susceptible to whitewashing, and there is controversy over whether text tone and forward-looking information have incremental information value [9-14]; (2) at present, there is no study that uses the readability of MD&A texts for early warning of company financial crises; (3) there is no relevant research on the Chinese market using the MD&A text similarity indicator[15]; (4) the financial crisis early warning methods for listed companies used in existing studies are mostly comparative studies using a single model or a few models, and there are relatively few comparative studies using multiple machine learning models [16-19].

The main contributions of this paper are: (1) Based on the same sample dataset, this paper measures the enhancement effect of each text-linguistic feature indicator in MD&A text on the financial crisis early warning of listed companies, which can provide a clearer picture of the incremental information contained in different text-linguistic features, and make up for the shortcomings of the existing research; (2) finding that the text readability and similarity indicators in MD&A text-linguistic features have incremental information, which is conducive to enhancing the accuracy of early warning models for listed companies' financial crises; (3) exploring the financial crisis early warning effectiveness of listed companies with the combination of MD&A text-linguistic feature indicators with enhanced early warning effect, which enriches the relevant research perspectives; (4) comparing thirteen mainstream machine learning models, and finding six of them with better and stable early warning effects, which provides a reference for subsequent model users.

**Point 3:** There is less introduction to web crawler technology in the manuscript.

**Response 3:** Thank you for your valuable suggestion. Considering that this article is not a technical article, the unoriginal web crawler technology is only a way to get the text, so it is not expanded to explain. In addition, considering that the existing manuscript already has a large amount of content, adding a detailed introduction to web crawler technology may lead to problems such as fragmented content and lack of focus, so we hope to have your approval and understanding.

**Point 4:** The annotation of the experimental results in the text is not clear, and it cannot be concluded which method is more superior. The conclusions obtained through experiments are limited in content and do not show particularly valuable conclusions.

**Response 4:** Allow us to explain further on this issue. Firstly, we introduce the evaluation indicators of the model early warning effect in the section "Machine Learning Model Early Warning Effect Evaluation Indicator", which are Accuracy, Sensitivity, Specificity and AUC value, and further introduce the meaning of the indicators and the specific measurement formula. In the subsequent empirical studies, the results of each sub-empirical study demonstrated the above four indicators, which are also specified in the empirical analysis. Specifically, when evaluating the financial crisis early warning effect of listed companies incorporating MD&A text-linguistic feature indicators, we use the average scores of the above four evaluation indicators of the 13 machine learning models to indicate the financial crisis early warning effect, and compare them with the effect of only the traditional financial indicators, in order to derive the value of the incremental information of the MD&A textual-linguistic feature indicators. In addition, we also compare the early warning effect of 13 models on different indicators of the same sample dataset to derive the recognition performance of 13 machine learning models for the samples. And the scores and rankings of the four evaluation indicators are shown in "Table A8. Mean values and ranking of early warning effect evaluation indicators of 13 machine learning models based on seven different sets of input feature variables", while are also discussed in section "Comparative Analysis of the Performance of Each Machine Learning Model for Sample Recognition".

Based on the above research, the paper draws the following conclusions:

(1) the addition of MD&A text tone and forward-looking indicators brings some noise and does not have a significant effect on the enhancement of listed companies' financial crisis warning effect; (2) MD&A text readability and similarity indicators both provide incremental information for listed companies' financial crisis early warning and improve the accuracy of that early warning; (3) the combination of text-linguistic feature indicators composed of MD&A text readability and similarity indicators is more effective in enhancing the early warning of listed companies' financial crises; (4) under seven different sets of input feature variables, RF, Bagging, CatBoost, GBDT, XGBoost and LightGBM maintain stable and accurate sample recognition, and these six models can be used as relatively optimal classifiers for listed companies' financial crisis early warning.

**Point 5:** The superiority of machine learning methods cannot be highlighted without comparison with other types of methods.

**Response 5:**

In this paper, based on the existing literature, we know that machine learning is a mainstream approach in the field of prediction with good predictive results.

Besides, this paper is not to emphasize that machine learning methods are superior to everything. After all, any method has its limitations. The focus of this paper is on the issue of the incremental value of information of MD&A text-linguistic feature indicators. Based on that, the issue of the superiority of 13 machine learning methods is compared.

**Point 6:** Some variables in the text are formatted in italics, while others are formatted in normal font. It should be unified. Please further standardize the format of the manuscript.

**Response 6:** Thank you for your valuable suggestion. The manuscript has been further adapted to the issues you have raised, as well as other formatting issues, in accordance with the style requirements of the journal.
