# Supplementary material for: Financial crisis early warning of Chinese listed companies based on MD&A text-linguistic feature indicators
Source: PLoS One. 2023 Sep 21;18(9):e0291818. doi: 10.1371/journal.pone.0291818 (PMC10513258; doi:10.1371/journal.pone.0291818)
Supplement: Attachment — Submitted filename: Response to Reviewer 4 Comments.doc [file pone.0291818.s005.doc]

# **Response to Reviewer 4 Comments**

**Point 1:** Is the manuscript technically sound, and do the data support the conclusions?

The manuscript presents an in-depth investigation into predicting financial crises in Chinese A-share listed companies, employing MD&A text readability, similarity, tone, and forward-looking indicators from 2011-2020. The authors claim that text readability and similarity are valuable for identifying financial crises, supporting existing research connecting these indicators to firm performance. However, they found that tone and forward-looking indicators did not enhance the identification of financial crises, as they can be manipulated by management. Utilizing machine learning models such as RF, Bagging, CatBoost, GBDT, XGBoost, and LightGBM, they provided a nuanced understanding of how combining text-linguistic features with traditional financial indicators can enhance early warning systems for financial crises. There are certain areas where the technical soundness could be improved, and where the data may not fully support the conclusions:

• Methodology Clarity: The methodology section could provide more detailed information about the data preprocessing, feature extraction, and the selection process for the machine learning models. Examples or specific rationale for the chosen methods would enhance the clarity.

• Control and Replication: The manuscript lacks adequate information about controls, replication strategies, and potential confounding factors, such as industry-specific patterns, economic conditions, etc.

• Sample Size Justification: A clear justification of the sample size would add to the rigor of the study and ensure that the findings are robust and generalizable.

**Response 1:** In response to **Methodology Clarity,** in addition to providing detailed explanations in the sections Evaluation Index System Construction, Sample Selection and Source of Data, and Data Processing and Descriptive Statistics in the paper, we have uploaded the code files and sample data to GitHub. The specific URL for accessing them is as follows. You can access them at the following URL: https://github.com/zzs0216/Financial-Crisis-Early-Warning-of-Chinese-Listed-Companies/tree/main (accessed on 19 August 2023).

In response to **Control and Replication**, allow us to provide further explanation. The paper does not study industry-specific patterns because its main contribution lies in measuring the enhancement effect of various text-linguistic feature indicators in MD&A texts on the early warning of financial crises, based on the same sample dataset. This study provides a clearer examination of the incremental information contained in different text-linguistic indicators, addressing the limitations of existing research. Furthermore, although industry-specific pattern analysis is valuable in the early warning of financial crises for listed companies, there are limitations when heavily relying on industry-specific analysis. These limitations encompass concerns regarding applicability, data availability, and model complexity. Specifically, (1) Applicability of general indicators: financial crises often exhibit a certain level of universality, extending beyond specific industries. Therefore, utilizing general indicators in financial crisis early warning can offer broader applicability and comparability. In contrast, excessive reliance on industry-specific analysis may restrict our ability to provide early warnings for other potential emerging industries. (2) Changes in industry specificity: industry-specific characteristics may undergo changes over time. A financial crisis early warning model for a particular industry may perform well, but its predictive ability could weaken with industry transformations or the emergence of new industries. Therefore, excessive reliance on industry-specific analysis may lead to neglect of industry changes or emerging industries. (3) Data availability: industry-specific patterns analysis relies heavily on a substantial amount of industry-specific data. However, for certain industries, the sample size of listed companies is small, which hinders the robustness of research conclusions. (4) Complexity of predictive models: introducing industry-specific patterns analysis increases the complexity of predictive models. This complexity can make the models difficult to comprehend and apply. In contrast, the use of general indicators is more straightforward and easier to understand, enabling their wider application in financial crisis early warning.

Indeed, based on our current study, future studies can further expand on industry-specific research, as well as the economic conditions you mentioned, such as economic booms, economic recessions, inflation, deflation, high unemployment rates, interest rate fluctuations, macroeconomic policies, market supply and demand dynamics, political risks, natural disasters, and various other economic conditions, to explore the early warning of financial crises in listed companies.

In response to **Sample Size Justification**, we have revised the section Sample Selection and Source of Data. The revised content is as follows:

Regarding financial crisis sample selection, scholars commonly consider a listed company being specially treated (ST) as a primary sign of being in a financial crisis [1-4]. Effective financial crisis warnings should have an early detection capability. Compared to the failure to meet debt obligations and bankruptcy, listed companies are often labeled as ST at an earlier stage. Thus, identifying ST status is more effective as an early warning. Therefore, this study selects companies in Shanghai and Shenzhen that firstly labeled as ST-listed from 2011 to 2020 as the sample for financial crises, totaling 481 companies over a 10-year period. The year in which a listed company experiences a financial crisis is defined as year T. ST refers to listed companies that have received special treatment for two consecutive years of losses, so the data in year T-2 can already show a financial crisis. To avoid overestimating the model’s prediction ability, this study utilizes data in year T-3 instead of year T-2, i.e., 2008 – 2017 data of both traditional financial indicators and text-linguistic feature. The selection of 2008 as the starting point is primarily driven by the implementation of new accounting standards for Chinese listed companies in 2007. This brought significant changes in the meaning and calculation methods of traditional financial indicators, resulting in lower comparability between data before and after 2007. Simultaneously, the global financial crisis erupted in 2008, leading to substantial changes in both the Chinese domestic and international economic environment. In response, China proactively provided new interpretations and adjustments to annual reports under the new accounting standards. This action enhanced the comparability of data after 2008. Additionally, the disclosure standards for MD&A texts were standardized after 2008, further contributing to the improved comparability of data after 2008. Therefore, based on these factors, we have chosen 2008 as the starting point for our research.

Subsequently, this paper excludes samples of listed companies in finance and insurance, as well as those with missing data on traditional financial indicators in year T-3 and missing text similarity. This results in a final sample size of 416 ST-listed companies. Considering the unbalanced number of ST-listed companies and non-ST-listed companies, this paper will explain the treatment in section Data Processing and Descriptive Statistics. The total number of processed samples is 832, comprising 416 non-ST-listed companies and 416 ST-listed companies. The traditional financial indicators data are obtained from the China Economic and Financial Research Database, while the MD&A text is crawled from the Cninfo website and processed into MD&A text-linguistic feature indicators through natural language processing technology.

**Point 2:** Has the statistical analysis been performed appropriately and rigorously?

The manuscript employs various machine learning models, but the statistical analysis needs further clarification:

• Model Validation: More details on cross-validation, hyperparameter tuning, and model evaluation metrics are needed to ensure the validity of the results.

• Comparison of Models: A thorough statistical comparison of the selected models against others would clarify why these specific models were chosen and how they outperform others.

**Response 2:** (1) About the detailed operation of cross-validation, this paper has a specific description, the content is as follows:

To enhance the generalization ability of the machine learning model and prevent overfitting, a 10-fold cross-validation method is employed in this study. This method divides the sample dataset into 10 copies, and takes turns to using 9 of them as the training set and 1 as the test set. The training set is used to train the machine learning model, while the test set evaluates its performance. To further increase the reliability and reduce result variability, a 10 times 10-fold cross-validation is conducted, and the averaged results serve as the estimation of the model’s effect.

(2) We have uploaded the relevant code for hyperparameter selection to GitHub, providing a clear understanding of the specific details of the hyperparameters. The specific URL for accessing the code is as follows: https://github.com/zzs0216/Financial-Crisis-Early-Warning-of-Chinese-Listed-Companies/tree/main (accessed on 19 August 2023).

(3) In the section Machine Learning Model Early Warning Effect Evaluation Indicator, we introduce the model evaluation metrics: accuracy, sensitivity, specificity, and AUC value. We further explain the significance of these indicators and provide their respective calculation formulas. In the subsequent empirical studies, the results of each sub-study displayed these four indicators, along with comprehensive explanations.

In response to **Comparison of Models**, please allow us to provide the following explanation. In the field of financial crisis prediction methods for listed companies, relevant research has evolved from univariate analysis to multivariate analysis and, more recently, the widespread adoption of machine learning methods. Therefore, this study investigates the predictive effectiveness of 13 mainstream machine learning models, which encompass traditional machine learning models, tree-based machine learning models, and ensemble machine learning models. It can be considered a comprehensive exploration rather than a specific one. We discuss the characteristics of these models in the section Machine Learning Models.

**Point 3:** Have the authors made all data underlying the findings in their manuscript fully available?

The manuscript does not provide complete information regarding the availability of the underlying data:

• Data Availability Statement: The Data Availability Statement should explicitly mention where the data can be accessed and whether there are any restrictions on data sharing.

• Data Description: A more thorough description of the dataset, including the web crawler technology used, and specific details about the extracted features, would enhance the reproducibility of the study.

**Response 3:** Thank you for your valuable suggestion. We have uploaded the data to the shared repository and it is described in Data availability and access. The description is as follows: the data and code of this study can be downloaded by visiting https://github.com/zzs0216/Financial-Crisis-Early-Warning-of-Chinese-Listed-Companies/tree/main (accessed on 19 August 2023).

In addition, we added descriptive statistics in the chapter "Data Processing and Descriptive Statistics", and the descriptive statistics of traditional financial indicators and text-linguistic feature indicators are shown in Table 2.

Table 2. Descriptive statistics of traditional financial indicators and text-linguistic feature indicators

| Variable | Obs | Mean | Std. Dev. | Min | Max |
| --- | --- | --- | --- | --- | --- |
| Earnings per share | 832 | 0.14 | 0.57 | -5.88 | 4.49 |
| Operating profit ratio | 832 | -0.21 | 4.18 | -116.76 | 13.97 |
| Debt assets ratio | 832 | 0.56 | 0.43 | 0.02 | 8.00 |
| Current ratio | 832 | 1.45 | 1.57 | 0.00 | 21.12 |
| Cash coverage ratio | 832 | 0.09 | 0.52 | -9.22 | 4.31 |
| Interest cover | 832 | 48.67 | 1,244.86 | -1,471.08 | 35,725.11 |
| Accounts receivable turnover ratio | 832 | 329.61 | 4,671.90 | 0.27 | 124,214.10 |
| Sales-to-cash ratio | 832 | 1.02 | 0.90 | 0.12 | 25.15 |
| Operating cash per share | 832 | 0.22 | 0.85 | -6.65 | 9.42 |
| Net profit growth rate | 832 | -131.71 | 1,301.70 | -23,572.69 | 13,737.70 |
| Tone Indicator | 832 | 0.40 | 0.17 | -0.22 | 1.00 |
| Forward-Looking Indicator | 832 | 0.01 | 0.00 | 0.00 | 0.02 |
| Readability Indicator | 832 | -21.88 | 4.26 | -42.82 | -11.20 |
| Similarity Indicator | 832 | 0.60 | 0.16 | 0.03 | 0.97 |
| ST (Not St is 1, St is 2) | 832 | 1.50 | 0.50 | 1 | 2 |

Regarding web crawler technology, we consider that this article is not focused on technical aspects. Therefore, the discussion of web crawler technology, which is not original, is limited to its role in obtaining text data without further elaboration. In addition, considering that the existing manuscript already has a large amount of content, adding a detailed introduction to web crawler technology may result in fragmented content and a lack of focus. Hence, we kindly request your approval and understanding in this matter.

Finally, the calculation methods for the ten traditional financial indicators and the four text-linguistic feature indicators are explained in the section Traditional Financial Indicators and the section MD&A Text-Linguistic Feature Indicators, respectively. The relevant data has also been uploaded to the public repository.

**Point 4:** Is the manuscript presented in an intelligible fashion and written in standard English?

The manuscript is mostly well-written but suffers from some grammatical errors and unclear expressions:

• Language and Grammar: Some sentences are complex and could be broken down into simpler structures to improve readability. A thorough proofreading is recommended.

• Figure and Table Descriptions: Additional explanations for figures and tables, along with concise captions, would enhance understanding.

**Response 4:** Thank you for your valuable suggestion. We have simplified complex sentences to improve the readability. We have also reviewed the manuscript for grammar issues and made necessary corrections. Moreover, we have revised the table captions and improved accompanying descriptions. The modifications have been tracked using tracked changes in the revised manuscript.

**Point 5:** Review Comments to the Author

This study presents a novel approach to early warning of financial crises in listed companies, leveraging text analysis and machine learning. While the concept is promising, there are several areas where the manuscript could be strengthened:

• Enhance the Technical Details: More information on methodology, statistical analysis, and data availability is essential to increase the rigor and reproducibility of the study.

• Consider Industry-Specific Analysis: Incorporating industry-specific characteristics might provide a more nuanced understanding and enhance the model's predictive power.

• Improve Language and Presentation: Proofreading for grammatical errors and enhancing figure and table descriptions will improve the manuscript's readability.

The addressed areas of improvement will not only enhance the technical soundness of the paper but also contribute to a broader understanding and applicability of the proposed methods. This research has the potential to make an impact, and I wish you all the best with its continued development.

**Response 5:** Regarding **Enhance the Technical Details**, in addition to providing explanations in the paper, we have uploaded the code files and dataset to a public repository to facilitate reproducibility and enable others to study and utilize them. As for **Consider Industry-Specific Analysis**, it can serve as a potential avenue for future research, and the specific reasons and explanations have been described in Response 1. Lastly, in response to **Improve Language and Presentation**, we have revised and improved the language and presentation.
