# Supplementary material for: Financial crisis early warning of Chinese listed companies based on MD&A text-linguistic feature indicators
Source: PLoS One. 2023 Sep 21;18(9):e0291818. doi: 10.1371/journal.pone.0291818 (PMC10513258; doi:10.1371/journal.pone.0291818)
Supplement: Attachment — Submitted filename: Reviewer comments.docx [file pone.0291818.s002.docx]

Reviewer comments

**Abstract：**

1. In the first sentence of the abstract, the author should provide a general overview of the background and importance of the study.

2. The part of the abstract that introduces the content of the article lacks logic. The author can use such logical words: "first", "on this basis", etc., to make the logic of the front and back more closely, and to help clearly express the point of view that the article wants to convey.

**Introduction**

1. It is necessary to cite literature to enhance persuasiveness. For example, which literature is referred to in this sentence: "Management's Discussion and Analysis (MD&A) contains further interpretation and analysis of the listed company's annual financial statements by the management"

2. In the part of introducing the background of the article, there is a lack of logic between sentences, and more consideration needs to be given to how to connect the sentences well.

3. Please describe the deficiencies of existing research in detail, and add corresponding references.

4. The contribution part of the article should be compared with previous research to enhance persuasiveness.

**Empirical Analysis**

The authors did not perform a descriptive analysis of traditional financial indicators. A descriptive analysis of the indicators should be added in subsequent revisions

**Related Studies:**

The literature review part is to summarize previous research. The literature review section does not need to elaborate on the research content of this paper again.

It is suggested that the author remove what was studied in this article.

**Conclusions:**

Please describe in detail what the inspiration of this article is.
