# Supplementary material for: Financial crisis early warning of Chinese listed companies based on MD&A text-linguistic feature indicators
Source: PLoS One. 2023 Sep 21;18(9):e0291818. doi: 10.1371/journal.pone.0291818 (PMC10513258; doi:10.1371/journal.pone.0291818)
Supplement: Attachment — Submitted filename: Reviewer comments round2.docx [file pone.0291818.s004.docx]

1，The author's manuscript still lacks literature support. Some of the views summarized by the predecessors need literature support. For example“Financial crisis early warning generally starts with selecting predictors that are indicative of whether a crisis will occur, and then applying statistical analysis methods or machine learning methods to identify the financial crisis.”Including but not limited to this sentence requires literature support.
2，“In terms of traditional financial indicators, this paper selects 10 specific financial indicators as benchmarks from five aspects metioned above to measure the performance of listed companies. In terms of text-linguistic feature indicators, this paper forms MD&A text tone, forward-looking, readability and similarity indicators for financial crisis early warning. ”This paragraph should be placed in the indicator description and construction section, not in the literature review. I have mentioned this suggestion before, please revise it carefully.
3，In the "Traditional Financial Indicators" section, please explain the reasons for selecting the indicators, rather than just explaining which literatures you refer to.
4，The Results of Empirical Analysis section only illustrates the model results. Authors are invited to moderately analyze the results.

5. Please polish your English expression.
